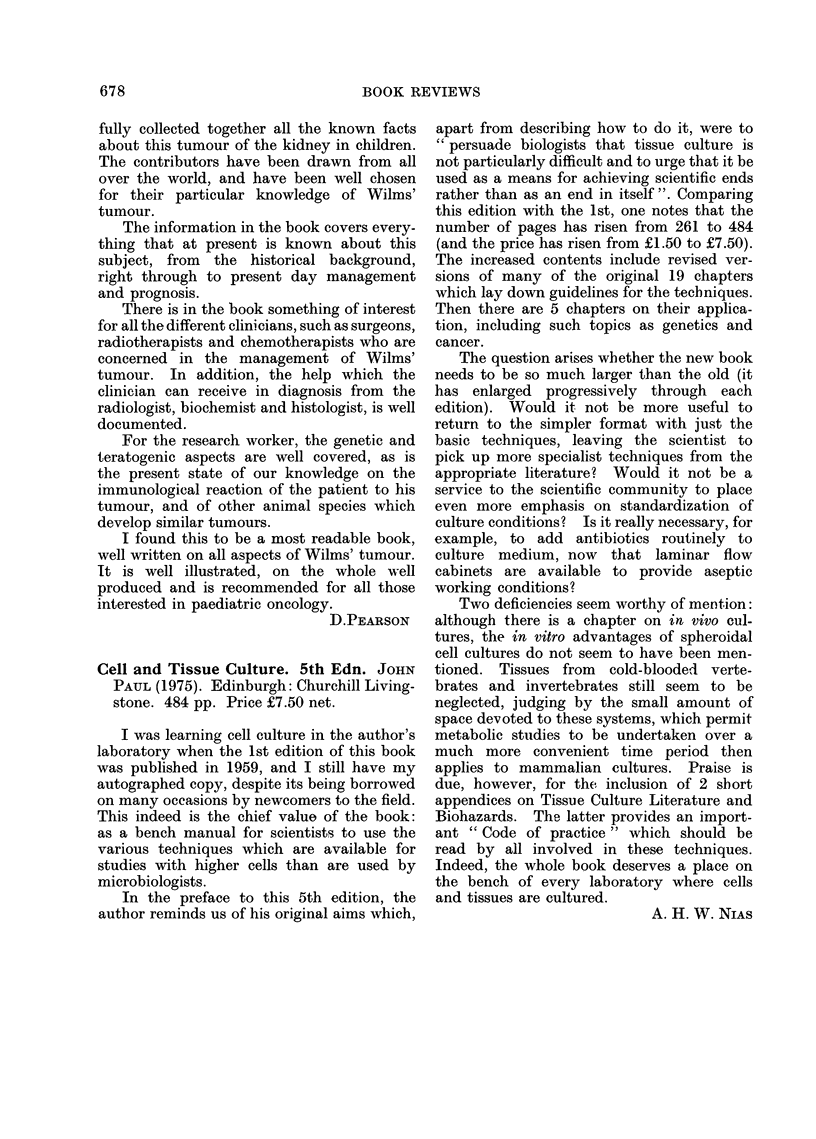# Cell and Tissue Culture. 5th Edn

**Published:** 1976-12

**Authors:** A. H. W. Nias


**Cell and Tissue Culture. 5th Edn.** JOHN PAUL (1975). Edinburgh: Churchill Livingstone. 484 pp. Price £7.50 net.

I was learning cell culture in the author's laboratory when the 1st edition of this book was published in 1959, and I still have my autographed copy, despite its being borrowed on many occasions by newcomers to the field. This indeed is the chief value of the book: as a bench manual for scientists to use the various techniques which are available for studies with higher cells than are used by microbiologists.

In the preface to this 5th edition, the author reminds us of his original aims which, apart from describing how to do it, were to " persuade biologists that tissue culture is not particularly difficult and to urge that it be used as a means for achieving scientific ends rather than as an end in itself ". Comparing this edition with the 1st, one notes that the number of pages has risen from 261 to 484 (and the price has risen from £1.50 to £7.50). The increased contents include revised versions of many of the original 19 chapters which lay down guidelines for the techniques. Then there are 5 chapters on their application, including such topics as genetics and cancer.

The question arises whether the new book needs to be so much larger than the old (it has enlarged progressively through each edition). Would it not be more useful to return to the simpler format with just the basic techniques, leaving the scientist to pick up more specialist techniques from the appropriate literature? Would it not be a service to the scientific community to place even more emphasis on standardization of culture conditions? Is it really necessary, for example, to add antibiotics routinely to culture medium, now that laminar flow cabinets are available to provide aseptic working conditions?

Two deficiencies seem worthy of mention: although there is a chapter on *in vivo* cultures, the *in vitro* advantages of spheroidal cell cultures do not seem to have been mentioned. Tissues from cold-blooded vertebrates and invertebrates still seem to be neglected, judging by the small amount of space devoted to these systems, which permit metabolic studies to be undertaken over a much more convenient time period then applies to mammalian cultures. Praise is due, however, for the inclusion of 2 short appendices on Tissue Culture Literature and Biohazards. The latter provides an important " Code of practice " which should be read by all involved in these techniques. Indeed, the whole book deserves a place on the bench of every laboratory where cells and tissues are cultured.

A. H. W. NIAS